# Training at the Gym, Training for Life: Creating Better Versions of the Self Through Exercise

**DOI:** 10.5964/ejop.v11i3.951

**Published:** 2015-08-20

**Authors:** Ceren Doğan

**Affiliations:** a Department of Psychosocial Studies, Birkbeck College, University of London, London, United Kingdom; Aalborg University, Aalborg, Denmark

**Keywords:** fitness, gyms, Re-Inventive Institutions, Total Institutions, thematic analysis

## Abstract

The present study draws on Scott’s (2011) notion of the Re-Inventive Institution and explores how gym members make sense and give meaning to their exercise regime. Overall, it is argued that for many participants gym exercise is more than physical training; it is also training for life. Based on a thematic analysis of 32 semi-structured interviews it is argued that gym workout is a means to create better versions of the self on mainly three levels. First, gym participants perceive themselves to be efficient and productive in general. Second, gym training is believed to increase the control they have over their lives. Third, gym members associate their gym workout with amplified emotional resilience, believing that fitness workout makes them not only fitter in a physical sense but also fitter and better equipped in a psychological sense. Surprisingly, a small group of regular gym users displayed more critical sentiments and distanced themselves from the images and values the gym stands for. The results of this study can be linked to broader political discourses on health and fitness that make use of corporate managerial vocabularies and are based on ideals of rationalization and efficiency.

## Introduction

Whether in the basement of a commercial complex, half-hidden between two corner stores, next to the tube station or on a wide green field in the suburbs, gyms have conquered urban space. Every European and North American city, and even small towns, seem to have a fitness gym. Gyms are one of the most pursued leisure places in Western societies and can be said to have established themselves as part of a white, middle-class culture (Featherstone, 2010; Howson, 2013; Phillips, 2005; Sassatelli, 2010; Shilling, 2005, 2008, 2012; Stebbins, 2009). In the UK, for example, almost 13% of the UK population is registered as members of a private health and fitness gym or a publicly-owned fitness facility, with London having the most registered users (European Health & Fitness Association, 2014).

Fitness gyms vary in location, membership fees and serve different social and economic milieus. Most urban gyms are located in the city centre and are at their busiest during lunch time and after work hours. In order to attract customers, most gyms offer more than a plain and functional working out environment but present themselves as lifestyle or family oriented places. Depending on the size and the target group, multi-purpose amenities encourage pre- or post-training activities, for example at their spas and beauty centres or they organize social activities at the weekend (Stewart, Smith, & Moroney, 2013). As Bryman (2004) notes, “hybrid consumption”, that is, consumption of several goods and services within one single place, tends to extend the time spent by the customers there. Consequently, one may think that the more time gym users spend at their gym, the more they engage with its material and social environment, and the more they are affected by the same so that the gym becomes more than just a training site for them.

## Literature Review

A substantive body of work on gyms deals with participants’ motivation to go to the gym (Crossley, 2006; Dworkin, 2003; Dworkin & Wachs, 2009; Laverty & Wright, 2010; Stern, 2008; Stewart, Smith, & Moroney, 2013). It is argued that one of the main motives is the desire to achieve a certain physique that conforms to contemporary aesthetic ideals (e.g. Dworkin, 2003). Crossley (2006) asserts, that for some participants the gym is an escape from everyday life where people can ‘turn off consciousness and submerse themselves in exercise’ (Crossley, 2006, p. 43). Laverty and Wright (2010) assert that going to the gym may provide individuals with a heightened sense of morality as going to the gym is in itself ‘a demonstration of desire to be a good citizen, to achieve and practice individual health responsibilities’ (Laverty & Wright, 2010, p. 79).

Drawing on the premise that femininities and masculinities are historically and culturally produced, the impact of fitness performances upon gendered identities has been addressed by various scholars (e.g., Craig & Liberti, 2007; Dworkin 2003; Heyes, 2007; Johansson, 1996; Johnston, 1996; McCreary & Saucier, 2009; Salvatore & Marecek, 2010; Tiggemann & Williamson, 2000). Dworkin (2003) writes that for most of its existence the gym has been associated with masculinity. The body building gym especially promoted and celebrated characteristics associated with male-ness, such as strength, power, competition and aggression, so that one could argue that through cultivating a muscular physical exterior, men were able to re-emphasize their superiority and dominance. Whilst this still may be true for bodybuilding gyms, contemporary fitness gyms seem to work in more complex ways. Women’s participation in gyms has widely increased and women entering the weight training area have become more common. Nonetheless empirical studies show that men and women tend to have very different objectives and motives for attending the gym (e.g., Haravon Collins, 2002; Salvatore & Marecek, 2010). Whilst male gym goers seem to be disproportionally concerned with arm, back and chest strength in contrast to lower body strength female participants are primarily interested in weight loss, and thus engaging more in cardio-vascular exercises.

As Featherstone (2010) puts forward, in contemporary Western societies, the body is understood as a reflection of one’s inner self so that one may argue that body modification technologies and body enhancement regimes can be understood as attempts to construct not only a beautiful, strong and fit appearance but also a beautiful, strong and fit self. One may then ask if people work out at gyms for more than body-related reasons, that is to say, if gyms also function as places in which people seek to alter and “re-invent” themselves in a more general sense.

## From Total to Re-Inventive Institutions

With her notion of Re-Inventive Institutions Scott (2011) draws on and expands Goffman’s (1961) concept of Total Institutions. In the following, I shall first outline Goffman’s conceptualization and then present Scott’s elaboration on it.

According to Goffman (1961), institutions fulfil certain functions in society. They serve either the majority ˗ the general public comprising normal, healthy and well-functioning citizens ˗ or they are designed to contain malfunctioning, deprived, sick or threatening minority groups. Schools, army barracks, work camps, and ships are only a few examples of the first mode of functioning whereas prisons, psychiatric hospitals, orphanages, retirement homes and hospitals are illustrative of the second. These localities are in some cases established to protect the majority from a threatening minority or, in other cases, to support an underprivileged population.

Goffman’s analyses are mainly concerned with the latter category and most notable, in the theorization and empirical study of what he terms Total Institutions. A Total Institution is ‘a place of residence and work where a large number of like-situated individuals, cut off from the wider society for an appreciable period of time, together lead an enclosed, formally administered round of life’ (Goffman, 1961, p. 11). Goffman describes different types of Total Institutions in which each of them can be qualified according to their function, degree of totality and mode of entering. By function he refers to the societal rationale of the institution (e.g. to care for the underprivileged, to contain those with an infectious illness, to protect society from dangerous others etc.). By totality Goffman refers to four common principles of institutional life: (1) a daily round ‘in the same place and under the same authority’; (2) activities carried out in the company of similar others; (3) timetabled activities that follow clear rules in the presence of designated officials; (4) scheduled activities that are part of a plan, designed to realise the goals of the institution (Goffman, 1961, p. 6). The last category of which Goffman speaks is the mode of entering, or, what may also be called the mode of recruitment. Access to a Total Institution can either be performed involuntarily with the prison being the typical case, or voluntarily. However, even where entering is a voluntary act it can nevertheless be subject to a selection procedure (see for example application procedures to high-ranking universities or monasteries). In any case, a Total Institution ‘can be viewed as a place for generating assumptions about identity’ (Goffman, 1961, p. 170).

Scott argues that in late modernity Total Institutions have become rare. The twenty-first century, she argues, is characterized by ‘institutions without walls’ (Scott, 2011, p. 3) that participants seek out to re-invent themselves on a voluntary basis. Examples of Re-Inventive institutions range from therapeutic clinics to spiritual retreats, academic hothouses, secret societies and virtual communities. Those institutions are characterized by members’ active engagement, by self-regulation and a desire to undergo deep personal change. In contrast to Total Institutions, they are often permeable, have flattened hierarchies and a cohesive inmate culture. As they are voluntary, and often costly there tends to be lack of overt resistance. High degrees of interaction amongst participants enable mutual surveillance and conformity to institutional norms. Indeed, as Scott argues, members of Re-Inventive Institutions actively look for out for other people’s company because they consider it pivotal to their own success, ‘wherein members gaze at each other and monitor their relative progress towards a shared role. This mutual surveillance implies a network of connections between inmates, who exercise an equally penetrating, ubiquitous gaze’ (Scott, 2011, p. 49).

Given that participants of Re-Inventive Institutions actively and willingly reproduce institutional discourses, one may consider the power through which the sense of self is transformed as qualitatively different from the power operating in Total Institutions where changes of the self are somehow motivated from without, and not necessarily from within.

Whereas traditional TI [Total Institution, author’s note] inmates were committed against their will, and new identities imposed upon them, now we find people choosing voluntarily to enter institutions, believing that they need to change, and that it is their responsibility to do so. (Scott, 2011, p. 2)

Scott suggests here that people seek out those institutions because they feel a responsibility towards changing and shaping their identities. This moral imperative, she further argues, stems from the fact that we live in what Furedi (2004) calls a *therapy culture*, a culture that calls for constant introspection and self-engineering in order to obtain happiness and personal satisfaction. Discipline and goal-orientedness is a pivotal element in organisations when the idea of success or progress is being emphasized. Progress can refer here to any category such as the physical, mental or psychological.

Scott’s concept is fruitful because it invites us to think of seemingly neutral, or innocent, practices and places as psychologically meaningful. Indeed, Scott’s own examples of Re-Inventive institutional sites are often everyday spaces on the boundary between work and leisure and may be easily overlooked. They involve ostensibly relaxing, even self-indulgent activities, pursed on one’s spare time and hence are prone to be regarded as irrelevant for people’s sense of self. However, each Re-Inventive Institution:

offers a different way of rethinking and transforming an incomplete self, and discursively produces different subjectivities (…) Individuals are encouraged to regard their fate as lying in their own hands, accept responsibility for their mistakes and free themselves from their shackles of deviant or unhealthy behaviour…Taking control of one’s own correction is viewed not as a punishment but as a privilege, a positive opportunity to boost self-esteem. (Scott, 2011, p. 98)

As stated above, members undergo re-inventive regimes not only because they regard it as a positive opportunity to boost self-esteem but also partly because they believe they have a moral responsibility to be healthy or to feel better. In the context of the gym one may then ask what participants hope to gain through the correction of their bodies and the advancement of their fitness levels, and relatedly, in which ways they feel incomplete or insufficient if they fail to do so. In this vein, the present study addresses the questions of how and to what extent the gym functions as an active and reiterative attempt to create better versions of the self. In other words, it asks to what extent gym participants seek to re-invent themselves other than on a physical level.

## Methodology

### Participants

The data for this study consisted of 32 semi-structured interviews with active gym members of whom 20 were women and 12 men, all students or working adults, ages 23 to 69. Respondents were recruited through a combination of personal contacts and snowball technique/referrals. The snowball technique itself has its limitations, self-selection being the most significant in the context of this project. The inclusion criteria for interviewees were minimal: 18 years old or over, English speaking, current gym membership and regular gym attendance with at least one gym workout session per week. Gym members who frequented the gym less often than once a week were excluded from this study. Members using the gym for other than exercise, such as the sauna or the Jacuzzi, were also not included to the data set.

### Ethics

This research project followed the recommended ethical guidelines of the *Birkbeck School of Social Science, History and Philosophy Ethics Committee*. All interviewees were afforded the right to anonymity and confidentiality. Whilst participants’ actual age and occupation are provided throughout the research report, every participant is given a pseudonym so that their responses cannot be matched to their personal details by anyone other than the researcher.

### Procedure

After establishing initial contact, by phone or email, and setting up a date, time, and location, interviews were conducted either in the cafeterias of their gyms or at a public place, at the respondents’ convenience. Each interviewee was provided with a form of consent that explained the rationale of the study, a confirmation of confidentiality and contact information. The interview schedule entailed questions about participants’ initial reasons to join a gym, the impact they considered their gym training had on their everyday lives and what they liked most about going to the gym.

### Interviewing Process

Initially an interview schedule was constructed in accordance with the research questions, the literature on gyms and the theoretical concerns of this project. After conducting three pilot interviews, some questions and issues were narrowed or expanded while others were changed substantially or abandoned altogether. Each participant was interviewed once; the shortest interview lasted 24 minutes and the longest 110 minutes. All interviews were audio-recorded and transcribed verbatim. I employed Braun and Clarke’s (2006) steps of theoretical thematic analysis for all transcripts, which means that the analytic procedure was driven by my theoretical and analytic focus. The benefits of thematic analysis is not only that it helps to identify, organise, analyse and report patterns (themes) within a data set but also its theoretical flexibility which means that it can be applied across a range of theories and epistemologies. In the following, I shall present the three key themes that I have identified as a result of this analytic process.

## Results

The results of the analysis revealed three key themes related to participants’ expectations and motives for exercising at the gym. Participants believed that exercise at the gym grants: a) more efficiency and productivity, b) a higher sense of control over their lives and c) an increase of psychological well-being. In addition, there was also a group of respondents who displayed (self-) critical sentiments towards the gym.

### Increasing Efficiency and Productivity

All participants agreed that the gym visit would positively affect the quality and “flow” of their day. Many talked about how they actually plan their gym visits in order to optimize this perceived effect. According to Rosie^i^ (age 24, post-graduate student), the gym gives her day a point of reference, a place either to depart from or arrive at.

Interviewer: When do you usually go to the gym?

Rosie: I like going [to the gym] in the mornings - I use the gym to structure my day. I get up around seven, then go to the gym for an hour or one hour and fifteen minutes. And then I will have my breakfast and a shower and get some work done between nine-thirty and ten. And I find when I don’t go, I get up later and then things move a lot slower. Like the process of going to the gym speeds up the day. It sets like a precedent to the way you want your day to go. Like if you get up and you have to do something, like going to the gym and you do it very quickly, it’s quite a motivating thing, running or whatever. Maybe it’s because maybe you are physically moving so far and everything is so fast, that sets a precedent and you are like wanting things to get done.

What is striking about Rosie’s account is the richness of details, describing how she follows a clearly structured morning routine split into different units of time. It indicates how carefully she organizes her day, making sure the gym is an integral part of it. The reason why she likes exercising in the morning before work is because it enables her to start and continue her day in a fast, efficient manner.^ii^ If she does not go to the gym, she feels that ‘things move a lot slower’. The gym, so it seems, accelerates other daily routines and sets ‘a precedent to the way you want your day to go’. Rosie reflects upon her own perception and hypothesizes that it might be related to the quick physical movements during the training that make her more driven over the course of the day. For Rosie, the gym regimen is strongly related to her work in-so-far as it creates a sense of efficiency. Indeed, the idea that the gym provides users with a sense of productivity, a sense of achievement that highly impacts the course of their daily lives in positive ways was a reoccurring theme in the interviews. A similar experience is captured in the following two quotations:

Rebecca: There is something kind of sluggish about the pace of your day unless you go. You kind of feel that things need to speed up at some point. Or you just sit around all day and sit on the tube, sit at work, sit at home, sit on the couch (…)

Boris: In life the urge to procrastinate is very strong and the gym helps me, becomes part of my program and for that, makes me more efficient (…) And since I’ve started exercising I am better at writing my thesis actually.

Whilst Rebecca (age 29, administrator) comments rather generally on how the gym responds to her ‘need to speed up’ things, Boris (age 27, full-time PhD student) refers to how the gym exercise increases his personal and professional efficacy. He has established the gym as part of his “program” by which he refers to his work schedule as a PhD student. Boris exercises at the gym not just to control and enhance his physical fitness but primarily to increase his work productivity. Such an approach to the gym was not exceptional in my sample. Many respondents, most notably young professionals, hold a similar view. One of the reasons why participants feel a higher sense of efficacy in their lives may be related to the ways in which gym reinforces ritualized self-discipline, something a large number of respondents referred to.

### Being Disciplined and in Control

As my interviews revealed, being disciplined during training is considered to be helping gym participants to be more disciplined outside the gym, as well. Gym practice was often described as something that may require extra effort and time, but it was often regarded as an investment that will ultimately decrease the effort and time that is needed for other things in life. When I asked Alexander (age 28, sales manager) whether he reported that the gym has an effect on his life in a broad sense:

Alexander: I find that you've started going to the gym and it's become part of your routine it gives you more energy to do other things just because you’re used to being active after a while and so all of a sudden say it's like ten at night and I need to go to the shop to get something, all of a sudden, I'm like yah, I'll just go and get it whereas beforehand, I'd be like, forget it I'll do it tomorrow, or another day or another day or another day.

As this quote illustrates, discipline and efficiency in- and outside the gym are thought to reinforce each other. The discipline, and perhaps rigour, gained at the gym is thought to be “contagious”, spilling over to other areas of life, bringing about an ease of handling everyday tasks.

Shawn: Going to the gym even though you're like spending loads of energy makes you a lot more disciplined in general I find. And it makes you just a lot more up for doing things in general. I like going to the gym because it keeps me going a bit more even when I'm not at the gym. And then you're probably a lot happier with yourself when you've been going to the gym a lot really. Whereas when I start going to the gym everything becomes more do-able.

Shawn associates the gym workout with being disciplined, efficient and ‘up for things.’ Crucially, he believes that the gym makes him happier, too, something that will be elaborated upon below.

Alexander: I think there's a hell of a lot of self-control that you achieve from going to the gym. I always think it's almost like you know how in like eating disorders you know it's so much about control which people never talk about in the media for some reason it's always about the self, the image that magazines are giving off and stuff and it's not, it's about control (…) I think it's about a feeling that going to the gym makes you feel. I get really proud of myself when I've been to the gym a lot in the week and done and feel really good about myself. You know it is sort of, a bit of it's not a challenge, well it is a challenge but not you know a sort of type of challenge which you do feel good when you pass your challenge.

Alexander reflects here on what might motivate him and other people to go the gym. He argues that aesthetic reasons are only one side of the coin, however, self-control is just as important. Pride and feelings of accomplishment are actually the ‘real’ reasons why people go to the gym, he believes. Popular discourse fails to see gym regime and eating disorders as closely related, he says, for both are based on self-discipline. Given that gym exercise is typically highly structured containing goals and sub-goals, it is likely for participants to see the gym as a challenge. Indeed, as the interview excerpts throughout this chapter have illustrated, feelings of accomplishment generated in the gym are not restricted to the locale but transcend its boundaries, leaving traces in the everyday life of its users.

One may also argue that discipline is inscribed into the gym’s material practices. What is common in all exercise equipment is a minute and precise utilisation, and management of bodily forces. The numerous calibrations, calorie counts, heart rate measures, repetitions and so on reveal one of the gym’s most significant logics, namely the utilization of optimum forces to effect self-improvement – a process that requires disciplining of bodies and minds and calls for the micro-management of movements, self-evaluating practices and self-rectification. Each machine sets little ‘challenges’ (weights, repetitions, sets, etc.) that users can either master or fail to master. Training at the gym, in other words, encourages a form of discipline that is directed towards the self. Gym members are complicit in the process of the disciplining their bodies; they learn how to work upon themselves according to given calibrations. This form of discipline elicits and fosters participants’ sense of accomplishment and gratification. In other words, because gym exercise is underpinned by a range of disciplinary procedures that primarily target the body and enhance its qualities, it evokes affective response related to self-mastery such as self-contentment, pride and enjoyment.

When I asked Meredith (32, client executive) how often she trains at the gym, she describes how her working-out pattern depends on the workload at her job:

Meredith: I try to go three to four times a week. It helps me to perform better at work (…) I exercise more when I am stressed. I certainly do, because - I certainly exercise more when I am stressed, I can make that correlation. It makes me feel as if I had a bigger sense of control in my life.

It seems that for Meredith exercise at the gym helps her to control her life. Although it takes time and effort to incorporate the gym into her daily schedule, she is convinced that the gym will ultimately help her to accomplish more and perform better in the realm of professional life. My analysis indicates that some participants regard it as a form of what might be called ‘rehearsal’, or training, for life; if one succeeds at this type of training, so the thinking of many participants goes, one will also succeed in other areas of life, become more productive and efficient. Personal qualities demanded by a serious fitness regime such as diligence, devotedness, and discipline, are expected to be echoed in the world outside the gym.

### Gaining Emotional Resilience

The majority of the interviewees mentioned a general quest for better health and fitness as their primary motivation to join a gym. However, there was a wide range of other issues participants expect to resolve, or tackle, with their gym regimen that go beyond fitness concerns. Matthew (age 31, part-time PhD student and IT manager), for example, described the gym’s benefits as follows:

Matthew: I sleep better, I eat better, yes, I look better, larger, bigger. I quite like it to be honest; it makes me more confident somehow. But this point is not as important as it was when I was twenty. It’s now more, I can feel that my testosterone level has increased constantly. And this makes me more confident, too. I’m more like ready to combat daily stress and I’m ready for situations where I need more power and stability. So the increased testosterone level is an important point for me in terms of the gym.

Being more confident, prepared, better equipped and resilient to external threats is regarded by him as a major gain of the gym. As he says, looking better, larger and bigger gives him the confidence and the ability to combat stress. Further, being ready for situations that require power and stability are, according to Matthew, achieved through the strong gym-shaped physique, and the increased level of testosterone. The firm, muscular body – so the thinking goes – not only projects but also causes psychological strength. The emphasis on the male sex hormone testosterone can be read at least as implying, if not openly stating, the supposed effects of exercise for the male sexual drive and potency. Matthew seems to imply that what one overcomes with exercise is ‘weakness’ and what one gains is power, stability and confidence, which can be translated into sexual energy and confidence, or, ‘manliness’. Indeed, the link between sexual potency and muscularity is continually being made in the world of fitness. One of the main strategies is to do so, is through the visual display of the muscly body. Shaved, smooth and tanned, the male body carries the aura of physical potency and yet goes further than that, implying sexual strength. Referring to the bodies of muscular public figures, Miles observes that “each [body] becomes a public phallus, huge, rock-hard, gleaming and veined with blood” (Miles, 1991, p. 111). In other words, the body’s ‘material’ qualities such as its contours, surfaces and textures become the locus of potency display. At the same time, it is implied that once one has all those physical qualities, one is also in possession of sexual power.

A further implication of the gym that was mentioned by interviewees is an expected increase in psychological well-being. Diane joined the gym to get fitter but also, as she explicitly states in the interview, to overcome a difficult time in her life.

Diane: Psychologically you feel better (…) I lost three of my closest relatives within four years. It was a difficult time - - And it’s good to do some exercise, to go out. It helps. Your brain – I don’t know much about it. The articles that I have read, it’s good for your body. Endorphins, isn’t it? (…).

At the time of the interview Diane (age 64, free-lance translator) has been a gym member for nine months. It was after the losses of some close family members that she decided to sign up. Following the advice in magazines, she hoped the gym would have therapeutic effects, helping her in this vulnerable life situation. Olivia (age 33, administrator) employed the gym also as a therapeutic strategy:

Olivia: Back then I had some serious problems with my job - and my relationship <laughing> Basically my contract had ended and with the recession and all that I had a really really tough time finding new employment. I don’t know how many applications I’ve sent out (…) I got seriously depressed. My GP wanted to prescribe some medication but I didn’t want that (…) That was when I gave the gym a thought, just to do something for myself and keep on going.

Instead of taking the antidepressants her General Practitioner advises her to take, Olivia decides to work out at a gym, hoping that the exercise will help her to “keep on going”. Given the recent break up with her boyfriend and the loss of her job she uses the gym as a ways to ‘pull herself together’. Both Diane and Olivia remain vague in their descriptions about how the gym workout exactly might have helped them to overcome a difficult time in their lives. Shawn (35, technical assistant) and Jessica (43, art historian) emphasize the gym’s positive effects on their psyche, too:

Shawn: It [the gym] blows off steam. You have a way of blowing off steam; you have a way of escaping. But not having that escape, I think allows frustration and depression and everything builds into you, it's not good.

Jessica: If I am not exercising at all, I am usually more stressed out and in worst moods. My stomach is more sensitive and upset to the foods I eat. I am like getting super bloated because all the stress goes to my stomach. … Physically I feel good because I take out my stress out in a workout; it doesn’t manifest itself in my stomach.

Stress, depression, bad moods and frustration are expected to be combatted by the gym regime. It is almost as if these negative states run the risk of manifesting themselves into the body (e.g. ‘the stress goes to my stomach’). Exercise, so the thinking goes, is purgative; it releases stress and frustration by relocating it to the exteriors of the body and soul.

### Critical Voices

Although most respondents emphasized the positive effects of the gym, a small number of interviewees expressed more ambivalent and self-critical sentiments. Interestingly, many of these critical voices came from respondents who are members of up-market fitness clubs but nonetheless wish to distance themselves from what their gym stands for.

Meredith: It’s mostly society saying that in order to fit in, or put your life together you need to have a gym membership just as you have a house, the best car, the spouse, the best children. If you are part of the really nice gym, like the one here, that has a spa and things like that. The more I talk about the gym the more I realize how much I hate it <laughing>

However, some interviewees commented critically on how their gyms seek to establish a link between the gym membership and a distinctive sense of identity - and how they had personally refused to take it up. Shawn, for example, is a member of an up-market club and describes the place as follows:

Shawn: The club is very sleek and cool. Some of the exercise rooms are decorated with purple lights, like a night club. The place promotes this kind of yuppie lifestyle. You have got the job, the apartment, you have the cool gym, you have this and that. But it’s all bullshit really. Most of these people don’t have cool jobs and all that.

Kate: The one I go to, it’s mostly young, professional crowd. I would prefer a gym that is not about how it looks, having the latest machinery and all, like aesthetically looking very cool, urban and hip but just like people that are just there to work out and aren’t focussed on images.

Alex: I think some people go to maintain image and appearance and this gym specifically promotes this nouveau, sophisticated very hip and cool and – I mean you walk into the gym and everything is white, it’s not functional at all.

The quotes above prompt us to think that gyms can function as re-inventive spaces in a sense that they that invoke the sense in people that they have bettered themselves and moved ‘up’ as well as ‘out’ of their own social class and thus obtained a more desirable social standing (Watt, 2007). By signing up to up-market gyms people may feel more urban, hip and, perhaps, as if they were wealthier and more sophisticated than they actually are. It might be worthwhile in future studies to explore why people continue go to the gym even though they ‘look through’ these strategies, that is to say, even though they experience a dissonance between what they do and what they think.

A few respondents also mentioned discomfort in relation to the ways in which trainers treat gym members.

Jennifer: Fitness trainers seem to want to arrive at where they are and the conversation seems to be ‘you must do this’, ‘you must push yourself’. I can push myself hard enough and I am not in this big competition in life. And this feeling I have always had with them was ‘work harder’, ‘work faster’, ‘push more’. I just want to get to this level. It might not be hugely ambitious but I’m ok with that. And I didn’t like the fact that they were always like. ‘C’mon I can do this, I can do this.’ And you are like, ‘ok but I’m not you’.

Kate: This whole motivational thing goes onto my nerves sometimes. ‘Run faster’, ‘Keep it up’, ‘Smile’. Really?!

Due to the limited scope of this study, the tensions between trainers and trainees could not be elaborated upon in more detail. However, the accounts above suggest that some participants may not follow the ambitions of their trainers, or the ethos of their gym. They may not want to “re-invent” themselves in the ways their trainers want them to, for example. To what extent people resist their trainers’ views or reject the idea of self-optimization that their gyms perpetuate must remain a topic for future research.

## Discussion

According to the results of this study, the gym can be said to be a Re-Inventive Institution first and foremost because of its, what might be called, “spill-over effects”. All respondents agreed that gym exercise has positive effects on other areas in life. Members expect the gym to optimize their work performance, their psychological well-being and ultimately their selves. As Scott (2011) writes, institutions that are concerned with physical appearance, beauty, fashion and ‘healthiness’ promote ideas and discourse that consume an actor’s consciousness throughout the day. Hence, one may argue that the impetus on self-discipline, self-optimization and on “becoming” becomes omnipresent for members’ also *outside* the gym.

Ritzer (1983) writes that a society characterized by rationality is one which values efficiency, predictability, calculability and control over uncertainty and puts a great deal of emphasis on finding the best or optimum means to any given end. This resonates with the results presented above. As interviewees state, fitness training at the gym can be considered a means by which transformation of and control over one’s life is achieved. One of the reasons why this may the case is that exercise in gyms itself requires disciple, self-surveillance and ambition. Indeed gym membership usually starts with the diagnostic procedure of a health check where weight, height, body fat, blood pressure, body mass index, etc., are measured and compared to what has been established as a scientific norm so that goals for further training can be identified and changes noticed. This may facilitate an experience of having control over one’s body and the power to alter. The gym, in this sense, can be said to be a path to perfection.

In his historical analysis of fitness gyms, Chaline (2015) writes that the gym has always been more than a place to train physicality. Ancient gymnasiums, as the author notes, were places for social interaction, recreation and leisure, but they were first and foremost educational institutions where the intellectual and athletic training of a military character was supposed to be accomplished. The Greek gymnasium, for example, was a popular recreational space for the members of the aristocratic class as it provided them with an opportunity to perform and enhance “one’s own outstanding persona and family” (Kah & Scholz, 2004, p. 14, translation of the author). The interconnectedness between physical training and personal development or self-optimization seems still to persist.

My results resonate with a particular stream in the literature on gyms that puts the idea of self-improvement, self-regulation and self-assessment at the center (e.g., Gill, Henwood, & McLean, 2005; Markula, 2003; Markula & Pringle, 2006). It has been noted that neoliberal understandings of health as a private matter increased people’s willingness to engage in ‘care of the self’ practices which lead to an increase of what may be referred to as the ‘body industry’ (Straughan, 2010). It is argued that the health and fitness industry is at pains to show how ‘imperfect’ bodies can be sculpted and corrected by the right diet, exercise and cosmetic products. Advertisements suggest that individuals are personally responsible for monitoring and controlling their bodies so that a slim and fit-looking physique does not only signify attractiveness but also self-control and ambition (e.g. Becker, 1993). In this context, it is also argued that the logics of paid labour have infiltrated leisure time in general and the relationship to one’s body in particular (e.g., Miller & Rose, 2008; Smith-Maguire, 2008a, 2008b). Baudrillard (1998) is one of the best-known advocators of this approach, arguing that the way in which individuals’ relations to their bodies is organized in a society mirrors the ways in which social relations and the relation to things are organized. He argues that private property and the accumulation of capital as the key tenets of capitalism are applied to the physical sphere, too: individuals understand their bodies as “things” that can be invested in, worked-upon and optimized. Fitness, according to this line of thought, is far from being playful or disengaged but a strategy to enhance the body’s qualities and value on the social and economic market, where it is surveyed and consumed by the gaze of the other (Frew & McGillivray, 2005; Sassatelli, 2010; Turner, 1999). The gaze of the other can be said to be crucial for people as it is linked to self-esteem. People strive towards other’s approval and recognition to enhance and regulate their self-worth (Brown, 2014). If one of the gym users’ motives and hopes is to create better, stronger and more resilient versions of their selves, as it is argued in this study, one may assume that there is a relative perceived lack thereof. The gym might then work as a place in which people work upon themselves to generate others’ recognition through self-enhancement, and therefore to increase their self-esteem. One may further speculate whether the correlation between physical exercise and self-esteem, that is repeatedly demonstrated in empirical studies, is indeed related to the fact that one gains social approval though physical fitness (e.g. Bowman, Cole, Dodsworth, Fenzi, & Burns, 2014; Joseph, Royse, Benitez, & Pekmezi, 2014).

One can argue that the logics of the free-market exceed the economic sphere and are internalized by individuals who go to the gym in a two-fold way: First, individuals allocate their time to the gym, that is to say, they choose to train there partly because they hope to produce a state of being that harmonizes with desirably attributes of the free market such as efficiency, productivity and emotional resilience. Second, the very ways in which gym participants engage with their bodies can be characterized in analogy to the working principles of the market. The division of the gym into separate areas as well as the different machines that address a limited number of muscles and not others, for example, strongly remind of the division of labour. To give another example, most machines “translate” bodily effort and time into calories and other numbers. This invites a way of thinking about the body as something that can be quantified, invested in and produced.

One may speculate what other forms of relating to the body and the self may be invented in future, not just in terms of fitness but perhaps also in terms of mental capabilities or even interpersonal relationships. The “quantified self” movement is perhaps the beginning of such a trend (Till, 2014). The use of digital self-tracking devices here takes the quantification of the body to its extreme: little detectors track what one eats, how one sleeps, how often one exercises, which friends one meets, how often one calls one’s parents, what books one reads and which emotions and physical reactions during these activities occur. These activities are then transformed into digital data, which are uploaded to servers that allow users to analyse their progress and share their information with other users (Till, 2014). As Lupton (2013) argues, the quantified self movement might be said to be an expression of neoliberal entrepreneurialism, celebrating self-maximisation and promoting self-critique through the presentation of “objective” measures of performance. To produce data about one’s self, to know one’s self better and eventually to improve seems to be one of the core tenets of contemporary society.

## Conclusion

This study has shown that the reasons for why people join the gym as an ‘institution without walls’ are manifold. To invoke Scott (2011), when people join institutions to alter themselves, it is often that they feel a personal desire and responsibility to create an optimized self. What participants expect to achieve at the gym is a better version of their selves in several ways. Firstly, many of the interviewed gym participants hope, and indeed perceive themselves to be more productive and efficient. Second, they feel they have more control over their lives when they train at the gym regularly. Third, they associate their gym workout with increased psychological resilience. It can therefore be said that these people engage in regular gym training to create a better, fitter and stronger version of themselves, that enables them to “keep on going”, to master their everyday lives, to cope psychologically with their stresses and strains.

Important to note, the sample of this study was limited for it consisted of students and working adults only, that is to say, of people who either sell or prepare to sell their labour force at the market. It would be interesting to explore how people out of employment such as retirees and other non-workers make sense of their gym exercise. Tulle and Dorrer’s (2012) study, for example, reveals that gym participants over 65 years old tend to come to the gym not only for physical training but also to form social bonds that exceed the boundaries of the fitness locale. In general, people who have been referred to the gym as a result of a medical condition might experience the gym differently than participants who come there for leisure, perhaps more as a compulsory “homework”. In an interesting case study, Nash (2012) shows that pregnant women do not only use the gym to get fitter for birth but also, quite paradoxically, to manage anxieties about weight gain. The author shows how pregnant gym users, more than non-pregnant women, compare the size and shape of their bellies to those of other pregnant women in aerobics classes. As she writes, the fact that body related anxieties manifest themselves in the embodied experience of group exercise challenges research suggesting that prenatal exercise has largely positive effects on mood and body image. For some of her informants, Nash maintains, pregnancy fitness means a third shift of work on top of their continuing commitment at home and in paid employment.

For the recruited participants exercise in a gym was obviously an important enough part of their lives for they volunteered and were interested to talk about fitness in general and their own fitness practices and histories in particular. It would be worthwhile to look at the motives of gym members for whom the gym is not that important or who train only occasionally as this may yield different results. Relatedly, it might be interesting to interview people who have strong feelings towards the gym because of their negative experiences there.

In future studies, it would be fruitful to explore to what extent gym-related practices resonate with broader discourses on health and fitness in a given society. It has been noted that in the last three decades or so, corporate managerial vocabularies have infiltrated governmental understandings and handlings of health with ideals of rationalization and efficiency, customer satisfaction, producer/consumer relations and performance targets (Numerato, Salvatore, & Fattore, 2012; Tonkens, Bröer, van Sambeek, & van Hassel, 2013). With the progressive abolishment of the social welfare state in many European countries, health is increasingly treated as a private responsibility and as something that one can purchase it might be worth discussing the gym as a social practice ˗ as an element of the commercialization of health services. Gyms can be said to speak directly to such neoliberal agendas, which try to increase the number of active and self-reliant citizens and to decrease the number of those who are dependent on the state and others. When gyms motivate their members to take responsibility for their own physical strength, they frame health as a feature of the self that individuals can and should responsibly manage. Taking on responsibility for one’s health has a normative and moral impetus, too, for lack of health ‘clashes too uncomfortably with the image of the “good citizen” as someone who actively participates in social and economic life, makes rational choices and is independent, self-reliant and responsible’ (Galvin, 2002, p. 107). Comparing and contrasting the results of this study with contemporary discourses and policies on health would help us to widen our understanding of not only participants’ motives to do gym exercise but also of the societal functions of gyms.
